# Depolarizing Actions of Hydrogen Sulfide on Hypothalamic Paraventricular Nucleus Neurons

**DOI:** 10.1371/journal.pone.0064495

**Published:** 2013-05-17

**Authors:** C. Sahara Khademullah, Alastair V. Ferguson

**Affiliations:** Department of Biomedical and Molecular Science, Queen's University, Kingston, Ontario, Canada; University of Toronto, Canada

## Abstract

Hydrogen sulfide (H_2_S) is a novel neurotransmitter that has been shown to influence cardiovascular functions as well and corticotrophin hormone (CRH) secretion. Since the paraventricular nucleus of the hypothalamus (PVN) is a central relay center for autonomic and endocrine functions, we sought to investigate the effects of H_2_S on the neuronal population of the PVN. Whole cell current clamp recordings were acquired from the PVN neurons and sodium hydrosulfide hydrate (NaHS) was bath applied at various concentrations (0.1, 1, 10, and 50 mM). NaHS (1, 10, and 50 mM) elicited a concentration-response relationship from the majority of recorded neurons, with almost exclusively depolarizing effects following administration. Cells responded and recovered from NaHS administration quickly and the effects were repeatable. Input differences from baseline and during the NaHS-induced depolarization uncovered a biphasic response, implicating both a potassium and non-selective cation conductance. The results from the neuronal population of the PVN shed light on the possible physiological role that H_2_S has in autonomic and endocrine function.

## Introduction

Hydrogen sulfide (H_2_S) is produced endogenously in mammals in a variety of different cells types from the brain, blood, skin, liver, and kidney [1]. It is considered to be the third gasotransmitter, along with carbon monoxide (CO) and nitric oxide (NO) [1], [2]. Since it was first observed to have non-toxic physiological effects in the brain by Abe and Kimura [3], the search to uncover alternative therapeutic roles for H_2_S has taken off. It has been implicated in the induction of long-term potentiation in the hippocampus, blood pressure regulation, inflammation, protection against oxidative stress, and the neuroendocrine stress axis [2]–[6].

H_2_S is produced by the action of one of three enzymes found in the mammalian body, cystathionine beta-synthase (CBS), cystathionine gamma-lyase (CSE), and 3-mercaptopyruvate sulfur-transferase (3-MST) [7], which biosynthesizes L-cysteine into H_2_S [8]. While not all aspects of endogenous H_2_S production are well understood, physiological sources of H_2_S include its production by enzymatic action, as well as release in response to physiological stimuli from stored pools in the cytosol and/or mitochondria [9]. These three enzymes have been suggested to be differentially distributed throughout the body, with CBS being highly expressed in brain tissue [3], [10], CSE in the liver, kidney, thoracic aorta, ileum, gastrointestinal tract, portal vein and uterus [11], and 3-MST localized to the mitochondria [12]. A source of non-enzymatically produced H_2_S appears to be inorganic polysulfides in red blood cells [13]. This ability for red blood cells to convert polysulfides into H_2_S allows for it to be delivered through dietary means [14]. While the physiological sources of H_2_S remain poorly understood, there are a few mechanism suggested in the literature.

Although difficult to measure endogenously, H_2_S concentrations in the brain have been estimated to be approximately 8 µM [15], while plasma concentrations have been suggested to be as high as 160 µM [1]. At these physiologically relevant concentrations, H_2_S has been found to play a role in vasoconstriction, as well as vasodilation. A study by d'Emmanuele di Villa Bianca and colleagues [16], showed that the application of a variety of NaHS doses (10 µM–1 mM) to the superior mesenteric artery produced vasoconstricting effects at low concentrations, while at high concentrations, NaHS caused vasodilation. Exogenous H_2_S has been shown to reduce blood pressure in various rat models [4], while Yang and colleagues [17], showed increases in blood pressure in CSE knockout mice. Studies observing H_2_S effects in the hippocampus showed that H_2_S enhanced NMDA receptor currents and facilitated hippocampal long-term potentiation (LTP) [18]. It was also found that NaHS abolished 6-hydroxdopamine (6-OHDA)–induced cell death and, as a result, rescued SH-SY5Y cells [19]. In the substantia nigra compacta, administration of NaHS eliminated 1-methyl-4-phenyl-1,2,3,6-tetrahyropyridine (MPTP)–induced dopaminergic neuronal loss [20]. In hypothalamic tissue, NaHS induces a concentration dependent decrease in the release of KCl stimulated corticotrophin-releasing hormone (CRH) [2]. Various ion channels have been implicated in these physiological responses to H_2_S, including ATP-sensitive potassium channels (K^+^_ATP_), large conductance calcium activated potassium channels (BK_Ca_), both T-type and L-type calcium channels, as well as chloride, transient receptor potential (TRP), and sodium channels (Nav 1.5) [6], [13], [21]–[23].

The paraventricular nucleus of the hypothalamus (PVN), located against the third ventricle within the hypothalamus, is a critical autonomic control center of the hypothalamus [24], [25]. More specifically, it has been implicated in central cardiovascular and volume control, including blood pressure regulation [26]. The PVN is also known for its role in the activation of the hypothalamo-pituitary-adrenocortical (HPA) axis through the secretion of the corticotrophin-releasing hormone (CRH) [27]. The PVN consists of three different cell types: magnocellular neurons (MNC), parvocellular preautonomic neurons (PA), and parvocellular neuroendocrine neurons (NE) [28], [29]. The magnocellular neurons project to the posterior pituitary gland and secret oxytocin (OT) or vasopressin (VP). The parvocellular preautonomic neurons project to the brain stem and the spinal cord and secret OT, VP, CRH, or thyrotrophin-releasing hormone (TRH). Lastly, the parvocellular neuroendocrine neurons project to the median eminence and secret CRH and TRH [30]. Recent evidence suggests that the PVN may act as a site of action for H_2_S. Microarray analysis has demonstrated that the three enzymes necessary to catalyze H_2_S are all present in the PVN and more recently, Gan and colleagues [31], found that microinjections of H_2_S into the PVN produce cardiovascular changes, although the physiological mechanisms through which H_2_S concentrations in the PVN might be regulated remain to be established. The purpose of the present study was to assess the role of H_2_S in the PVN and the downstream physiological effects it may have using patch-clamp recordings.

## Methods

### Slice preparation

Male Sprague-Dawley rats (Charles River, Quebec, Canada) aged 21–28 days were decapitated according to the regulations established by the Canadian Council on Animal Care and approved by the Queen's University Animal Care Committee. The brains were removed and placed in ice-cold solution composed of (in mM): 87 NaCl, 2.5 KCl, 25 NaHCO_3_, 0.5 CaCl_2_, 7 MgCl_2_, 1.25 NaH_2_PO_4_, 25 glucose, 75 sucrose bubbled with 95% O_2_/5% CO_2_. The region of the hypothalamus containing the PVN was isolated, and 300 µm coronal sections were cut using a vibratome (Leica, Nussloch, Germany). Slices were then incubated at 32°C for a minimum 1 hour prior to recording in carbogenated artificial cerebrospinal fluid (aCSF) containing (in mM): 126 NaCl, 2.5 KCl, 26 NaHCO_3_, 2 CaCl_2_, 2 MgCl_2_, 1.25 NaH_2_PO_4_, 10 glucose saturated.

### Electrophysiology

Slices were mounted onto the stage of the recording chamber, which was continuously perfused at a flow rate of 2–3 ml/min with carbogenated aCSF at approximately 32°C. An upright differential interference contrast microscope (Scientifica, Sussex, United Kingdom) at 40× magnification was used to visualize the neurons. Using a Sutter Instruments P97 flaming micropipette puller, borosilicate glass electrodes (World Precision Instruments, Sarasota, Florida, USA) were pulled and filled with an intracellular solution composed of (in mM): 125 potassium gluconate, 10 KCl, 2 MgCl_2_, 0.1 CaCl_2_, 5.5 EGTA, 10 HEPES, 2 NaATP (pH 7.2 with KOH). Electrodes were optimized to have a resistance of 3–5 MΩ. Whole cell recordings were made with a Multiclamp 700B amplifier (Molecular Devices, Sunnyvale, California, USA) and acquired at 10 kHz, and filtered at 2.4 kHz using a Micro 1401 interface. Once a high resistance seal was obtained, whole cell configuration was accomplished by breaking through the cell membrane to gain access to the internal contents of the cell through the application of a brief period of negative pressure. Only neurons with a minimum spike amplitude of 60 mV (range 60–100 mV), input resistance of >400 MΩ (range 400–1100 MΩ), and series resistance of <20 MΩ (range 8–20 MΩ) were included in our subsequent analysis. The data was recorded in Spike2 software for offline analysis (Cambridge Electronic Devices, Cambridge, UK). PVN neurons were characterized as MNC (delayed return to baseline in response to a hyperpolarizing pulse), PA (calcium spike in response to a hyperpolarizing pulse), or NE (neither of the above) using a standard current pulse protocol prior to the application of NaHS [28]. Following a minimum 100 s baseline recording period, specific known concentrations of sodium hydrosulfide hydrate (NaHS) were applied to slices through a bath perfusion system. The response to NaHS was determined by comparing the mean membrane potential of the neuron before and after application and averaged across 100 second time periods. A response was considered significant if the change in membrane potential after NaHS application was at least twice the amplitude of the standard deviation of the baseline membrane potential during 100 seconds prior to application. Descriptive statistics were also used to describe mean group data as well as the standard error of the means in all such groups. The recorded membrane potential was adjusted to correct for the calculated junction potential (−15 mV).

### Chemicals and drugs

All salts used to prepare the slicing solution, aCSF, and the internal recording solutions, NaHS were obtained from Sigma-Aldrich Pharmaceuticals (Oakville, Ontario, Canada).

## Results

### Hydrogen sulfide influences the excitability of PVN neurons

**Figure 1:**
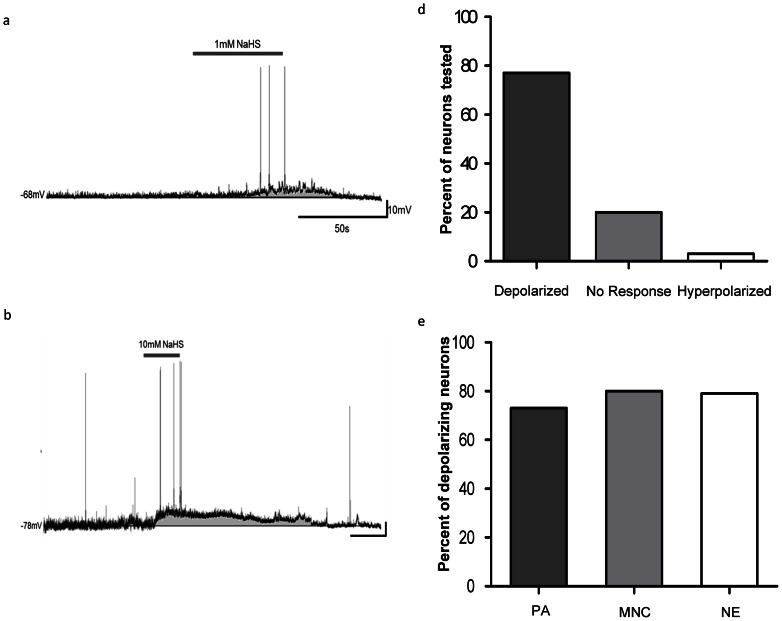
Hydrogen sulfide depolarizes PVN neurons. Traces illustrate the depolarizing effects of NaHS application on PVN neurons. a) Current clamp recording trace illustrating a depolarizing response to 1 mM NaHS. Trace b) shows a current clamp recording illustrating a depolarizing response to 10 mM NaHS. Bar graph c) illustrates the various responses to NaHS (0.1–50 mM) of PVN neurons (80%, n = 52/65 depolarized, 20%, n = 13/65 showed no response, and 3%, n = 2/65 hyperpolarized). Bar graph d) shows the percentage of PA (73%, n = 24/33), MNC (80%, n = 12/15), and NE (79%, n = 11/14) neurons that depolarized.

**Figure 2:**
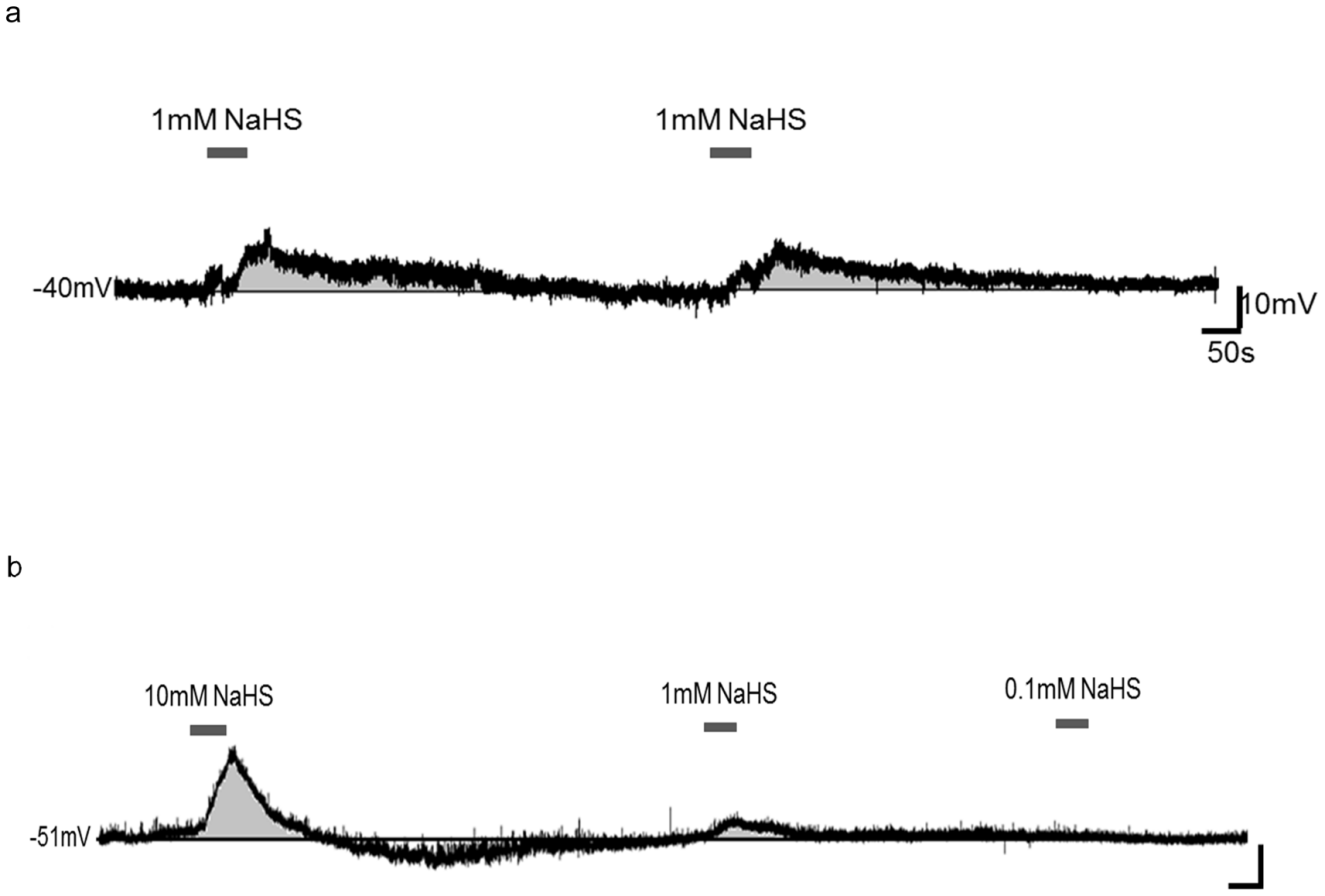
Hydrogen sulfide has reproducible responses and effects the various cell types in a similar manner. a) Current clamp recording trace illustrating a PVN neuron's rapid and repeatable response and recovery to 1 mM NaHS. Trace b) shows various concentrations (10 mM, 1 mM, and 0.1 mM) applied in the same neuron.

**Figure 3:**
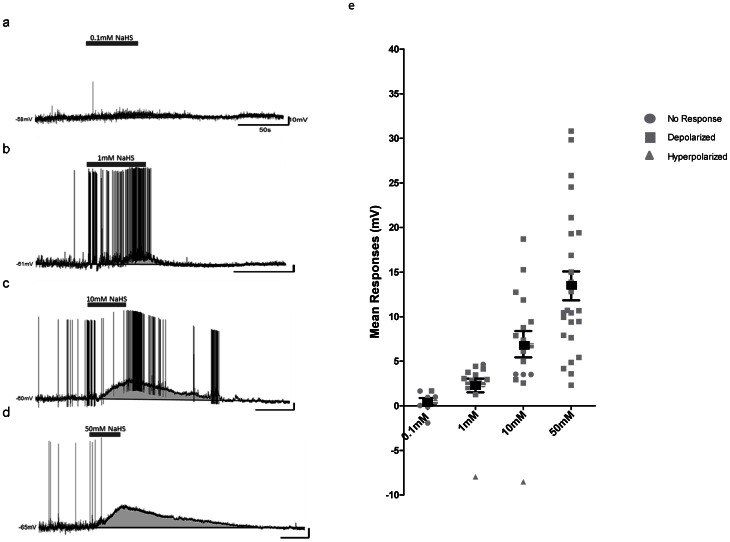
Hydrogen sulfide has a concentration dependent relationship. Traces illustrate the magnitude of the depolarizations in response to the various NaHS concentrations (0.1, 1, 10, and 50 mM). As the concentrations increase so does the response. a) Current clamp recording trace illustrating no response to 0.1 mM NaHS. b) Current clamp recording trace illustrating a depolarizing response to 1 mM NaHS. c) Current clamp recording trace illustrating a depolarizing response to 10 mM NaHS. d) Current clamp recording trace illustrating a depolarizing response to 50 mM NaHS, with an increase in firing frequency during the initial phase of the depolarization. e) Scatter plot showing the response of all recorded neurons to the various NaHS concentrations (0.1, 1, 10, and 50 mM) with the mean response and standard deviations indicated by the black square and bars.

**Figure 4:**
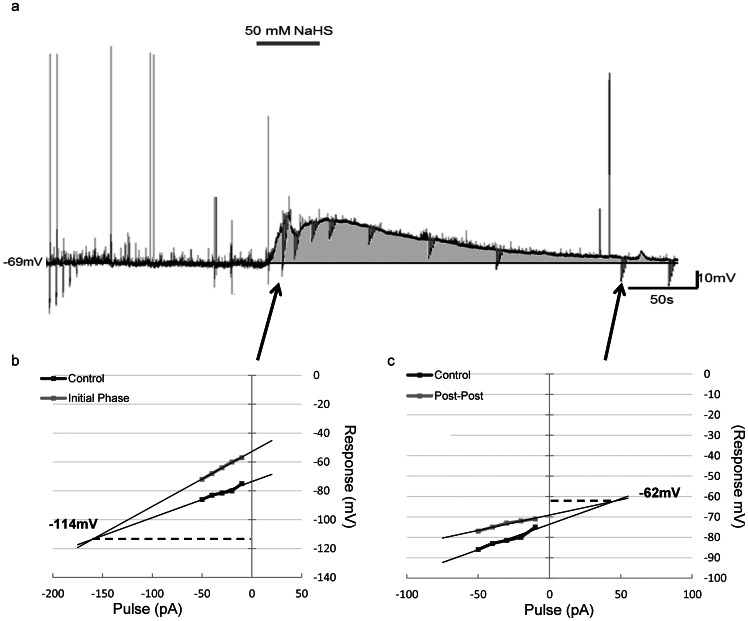
Hydrogen sulfide-induced depolarization is biphasic. a) Current clamp recording trace illustrating the change in input resistance over the duration of the hydrogen sulfide-induced depolarization (50 mM). b) V/I plot showing the change in input resistance (point of intercept, 114 mV), during the initial phase of the depolarization. c) V/I plot showing the change in input resistance (point of intercept, 62 mV), during the recovery phase of the depolarization. Input resistance calculated from the slope of the line.

Hydrogen sulfide was bath applied to a total of 65 PVN neurons during current-clamp recordings. The majority (n = 52, 80%) of neurons were responsive, with (96%) of responsive cells depolarizing, which was characterized by a rapid rise in membrane potential immediately after NaHS entered the bath, followed by maintenance of the effects for the duration of donor application, and a rapid recovery to baseline membrane potential associated of replacement of bath solution with aCSF (Fig. 1). The effects of NaHS were reproducible, in that when the same concentration was applied for a second time while recording from the same neuron, a similar second response was observed as illustrated in Figure 2a. The depolarizations were usually accompanied by an increase in firing frequency during the initial rise in membrane potential (Fig. 3b, c, and d). The remaining 2 (4%) cells influenced by NaHS responded with a rapid, long lasting hyperpolarization followed by a return to baseline. The depolarizations also appeared to be mediated by more than one ion channel as shown in Figure 4.

### Hydrogen sulfide exerts similar effects on MNC, PA, and NE PVN neurons

We also examined the effects of hydrogen sulfide on PVN neurons differentially classified according to their electrophysiological fingerprints as MNC, PA or NE. NaHS influenced the membrane potential of PA neurons (73% depolarize, 27% no response, n = 33), MN neurons (80% depolarize, 13% hyperpolarize, 7% no response, n = 15) and NE neurons (79% depolarize, 21% no response, n = 14) (Fig. 2b). After categorizing the neurons according to these criteria, it appears that NaHS does not influence the distinct cell types in a different manner and therefore all cell types were grouped together for analysis.

### Hydrogen sulfide affects PVN neurons in a concentration-dependent manner

The depolarizing effects of NaHS were also found to be concentration dependent as illustrated in Figure 3e. Bath application of 50 mM NaHS elicited a depolarizing (mean 13.46±1.62 mV) response in 100% (n = 25) of the recorded neurons, followed by a return to baseline membrane potential which usually occurred within 60 s as illustrated in Figure 3d. We also examined the effects of lower concentrations of NaHS, with 10 mM influencing 79% (n = 11/14) of neurons tested with 10 of 11 showing depolarizations (mean 10.38±1.34 mV) and similar recovery times, as illustrated in Figure 3c and e, while the 1 cell which hyperpolarized (−8.53 mV) showed a much longer recovery time of nearly 10 minutes. A similar pattern of responsiveness was seen following 1 mM NaHS with 83% (n = 15/18) of neurons responding, with the majority (n = 14) of neurons showing much smaller depolarizing effects (3.00±0.28 mV), and similar short recovery times which can be seen in Figure 3b. Again 1 cell hyperpolarized (−8 mv) again with a longer recovery time of 3 minutes. Bath application of 0.1 mM NaHS failed to elicit a response in 7 of the 8 cells tested as illustrated in Figure 3a, although 1 neuron responded with a small depolarization (1.7 mV). Overall, the vast majority of the PVN neurons tested responded with depolarization and the concentration dependent nature of these effects are summarized in Figure 3e.

### Hydrogen sulfide-induced depolarization is mediated by multiple ion channels

In order to identify potential ion channels responsible for the NaHS-induced depolarization, we applied hyperpolarizing current step protocols to the neurons to examine the changes in input resistance. This hyperpolarizing pulse protocol was applied prior to the application of NaHS, immediately following the application (initial phase), and as the membrane potential was recovering back to baseline from the effect (recovery phase). Voltage/current (V/I) plots were generated for all five points and demonstrated as illustrated in Figure 4 that NaHS resulted in an initial large increase in input resistance (control vs. initial phase) during the initial phase of the depolarization, as seen in Figure 4b, followed by a large decrease (control vs. recovery phase) during the recovery phase which often lasted beyond a return to baseline membrane potential (Fig. 4c). The point of intersection of these voltage current plots also provided information about the reversal potential for ion channels potentially modulated by H_2_S. This point was calculated from the slope of the control line versus the slope of either the line from the initial phase or from the recovery phase. The intersecting points were then averaged for each group (initial phase vs. recovery phase). This analysis suggested the initial stage of the NaHS-induced depolarization may result from the inhibition of potassium channels as the reversal potential was −103.6±8.4 mV (n = 5) (Fig. 4b). Conversely, the point of intersection taken from voltage current plots during the recovery phase (−53.6±9.8 mV, n = 5) indicated the involvement of a non-selective cation current as illustrated in Figure 4c.

## Discussion

In this study we have used *in vitro* whole-cell patch clamp techniques to show that the H_2_S donor, NaHS increases the excitability of the majority of neurons within the PVN as shown in Figure 1d, suggesting important roles for H_2_S in the regulation of diverse neuroendocrine and autonomic function [24]. We observed that the H_2_S donor caused rapid onset and reversible depolarizations in the vast majority of PVN neurons tested as illustrated in Figure 1 a, b, and c, these effects were similar to those previously reported in dorsal raphe serotonergic neurons and oxygen sensing cells in trout gill chemoreceptors neurons [32], [33]. Nagai and colleges [34] reported that H_2_S produced an increase in intracellular calcium (Ca^2+^) concentrations and Ca^2+^ waves in cultured astrocytes and hippocampal slices, suggesting potentially important interactions between glia and neurons, interactions which could contribute to depolarizing responses [35]. The depolarizing effects appeared to be concentration dependent with only 1 neuron responding by depolarization to 0.1 mM NaHS, with increasing magnitude depolarizations following 1 mM, 10 mM, and 50 mM which was the highest concentration tested in our studies (Fig. 3). Similarly, Umemura and Kimura [36] reported that H_2_S has dose-dependent responses on the reduced metabolic activity in rat neuronal cultures and neuroblastomas. A concentration dependent response was also seen in hypoglossal rootlets of medullary brain slices, in which there was increased activity as the H_2_S concentration was increased [37].

According to the definition given by Abe and Kimura, [3], the lower concentration used (0.1 mM) falls within the physiologically relevant range, which is similar to the endogenous H_2_S concentrations reported in brain homogenates (∼160 µm). It is important to note that since H_2_S is a fairly volatile and an unstable gas, the concentrations that were stated and desired may not have been the final concentrations to reach the cells [38]. According to Olson [38], approximately 13% of dissolved H_2_S is lost with every minute of application, and the loss continues to increase when the solution is oxygenated. Therefore, higher concentrations of the donor may be necessary to elicit a response [38]. When dissolved in aCSF at 37°C, H_2_S has a pH of 7.6 [39]. While we measured the lower concentrations (0.1 mM, 1 mM, and 10 mM) to be within close range of what can be considered physiologically relevant by definition, it should also be noted that both the pH and the osmolality measures across all of the concentrations except for the highest (50 mM) were within physiologically acceptable ranges [40], [41].

Also, of importance is the ability of NaHS to produce such rapid responses and recoveries of the membrane potential in the PVN neurons, as illustrated in Figure 2a. The rapid neuronal response produced by H_2_S can translate into rapid onset and inhibition of physiological effects. These results are comparable to the results obtained in the carotid body in response to H_2_S administration [42]. With that said, while the neurons were able to rapidly recover back to baseline, the integrity of the cell may not have fully recovered. It appeared that for an extended period of time after the neuron had recovered and achieved the pre-NaHS administration resting membrane potential it showed a decrease in input resistance, implying that several ion channels remained open (Fig. 4a).

The present work is the first to suggest that H_2_S acts to modulate two different ion channels as summarized in Figure 4. We observed different reversal potentials for effects on input resistance immediately after H_2_S application when compared to the later period during recovery to the control membrane potential. Our reversal potential data indicates that NaHS in the PVN is modulating a potassium current immediately following administration, indicated in Figure 4b, however, as the neuron's membrane potential recovers toward baseline a separate conductance with a reversal potential in the range of a nonselective cation conductance is apparently activated (Fig. 4c). While our data implicates both potassium and non-selective cation channels in the depolarizations produced by NaHS, future voltage clamp studies will be necessary, both to confirm such actions on these specific conductances, as well as to more clearly define the membrane events underlying such interactions. In accordance with our observations potassium channels have been implicated as one of the primary targets through which H_2_S exerts its vascular effects to produce changes in blood pressure and other cardiovascular variables [43]. In the central nervous system, H_2_S has also been implicated in altering intracellular potassium levels which are known to regulate cellular apoptosis, through effects on osmolality, caspase activation, and mitochondrial activity [44]. A recent study observing the effects of H_2_S in the brain, have shown that not only can it rescue the brain from cerebral hypoxia in a concentration dependent manner, it does so through a potassium channel [45]. A number of other groups have associated H_2_S elicited responses with numerous nonselective ion channels, showing that H_2_S can modulate its effects through various TRP channels to produce anti-inflammatory responses [46].

Our data also shows that the administration of NaHS produces consistent responses among the MNC, NE, and PA neurons within the PVN, as illustrated in Figure 2b. These cell types send projections to different brain regions including the brain stem, anterior pituitary, and the median eminence to mediate physiological autonomic and endocrine functions [24], [28]. This finding provides some evidence that H_2_S may have implications on several core autonomic and endocrine functions, such as, circadian rhythm, cardiovascular function, the stress axis and fluid balance, among others, as a result of the consistent depolarizations observed across all PVN cell types by the administration of NaHS. Some evidence for this was observed by another group which demonstrated an H_2_S concentration dependent decrease in potassium chloride stimulated CRH release from rat hypothalamic explants, which downstream inhibited stress-related glucocorticoid release [2]. Most recently, Gan and colleagues [31], observed increases in renal sympathetic nerve activity, mean arterial pressure and heart rate in response to NaHS microinjections into the PVN, further indicating that H_2_S has an effect on the physiological functions produce by the PVN.

Our electrophysiological studies of the effects of NaHS on neurons in the PVN are the first to show that this neuromodulator exerts direct actions on the excitability of this neuronal population. Our results were also able to shed light on the possible ion channels involved in NaHS modulation in the PVN, and provide insight into the possible role of NaHS in autonomic and endocrine function.
